# History of Dentistry

**Published:** 1889-03

**Authors:** 


ARTICLE II.

### HISTORY OF DENTISTRY.

The November meeting of the Rochester Dental Society was held at the office of Dr. W. F. Arnold, who entertained the Society with the following paper:

History is ever repeating itself in different forms. Since the human race has reached in its evolutinary development up to the eruption of teeth, some kind of dentistry has been needed.

Dentistry is named from the Latin word dens, a tooth.

There are shells and plants named dental from having points in structure like the tooth.

Dental Surgery, so far as we have been able to learn up to the present century, was a very crude performance.

And the theory of dental caries equally crude and amusing. The care of the teeth was but little known and what literature we had on the subject was very entertaining from its embryotic condition.

Ambrose Pare said in his treatise on surgery that a man should so carry himself that he did not in pulling teeth pluck out two teeth for one.

While Prof. Darby was traveling in the East he had a Bedowin Sheik walk fourteen miles to have a tooth extracted by him. He sat on a keg outside of the professor's tent and had the operation performed, and really seemed to enjoy it. The custom in that country among the tribes is to have the muleteer perform that branch of surgery; in which they become quite skillful. They take a mallet and a chisel. The patient opens his mouth and the dragoman puts his punch or chisel down close to the gum and drives out the tooth with a smart blow of the mallet.

In China tooth pullers are trained from early youth to do it with their thumb and first finger. They are taught to pull nails out of boards and by this continued practice get great strength of muscle, and perform this surgical operation very dextrously. Much of their medical and surgical knowledge is not up to this standard. Persons suffering with pains in their head or face will go to one of the native doctors, who, for the most part, are sorcerers or magicians, who interpret dreams, tell fortunes and find stolen goods. He will tell his patient that there are worms in his brain or head, and that they must have a chance to crawl out, so that he will have to make a hole in the head, either through the ear or eye, and whatever organ the person so applying for relief chooses is punctured usually without any relief, so that in China many are deaf and blind.

In 1541 a little book was published with XIII.

chapters, on the care of the teeth. One statement is, that few have, instead of teeth, a whole bone to chew with. It states that in old age people lose their teeth and new ones grow. It give an account of Zankelin, who got some teeth at the age of one hundred and four years. And it further says, his brother never lost his and did use them up completely. I will state. that I, several days ago, removed the roots of a whole set of teeth from the mouth of an old lady who had used her's up so completely that not a crown was left on any root. I should judge she had never had a tooth pulled, but let one after another decay down to the gum, till it was necessary to remove them on account of their ragged edges irritating the gum and causing inflammation. The authority I have just spoken of says that vomiting injures the teeth. Also hot victuals burn the teeth and can not be taken without hurt; also, never go to sleep after a meal for this damages the teeth. In speaking of teething, the author says children's gums should be bathed and rubbed with chicken, goose, or duck fat, or the brains of a hare may be taken and rubbed over the gums, mixed with the fat spoken of, and after this wash the head and face, also the neck with tea made from melilot, mallows, chamomile, cubebs. When the teeth break through a little wool from under the neck of a sheep nicely dipped in cubeb oil or chamomile laid warm on the gums and jaw.

He also teaches when the first teeth drop out the new ones should be pressed into the place of the old till it sets there among the others.

On Odontalgia. The same author says: "No one knows what toothache is better than he who has had it. Cure is to bleed and cup and to open veins such as the cephalic or those under the lips and tongue, or purging; or take the skin of a snake, boil it in vinegar and hold it in the mouth, that is very good. There are many other things equally good as those named which your time and patience will not permit, as I must hasten on to the main points in the history of our profession."

Heroditus first mentions dentistry (500 B. C.). The Egyptians were the most advanced. Teeth filled with gold have been found in the mumy pits. A collection in Liverpool contains specimens of artificial teeth, and two teeth of Sycamore wood set in gold. It is thought by some that it is only a kind of decorative gilding done in honor of the dead.

Celsus lived about (100 B. C.) and first recommended the use of the file for removing decay and sharp projections on the teeth. After this writer little is said about the teeth until the fifteenth century, and from that on to the present time. The latter part of the eighteenth century the anatomy and physiology of the teeth was very nearly as well known as it is now.

In 1774 and 1776 mineral teeth were first introduced into France. Previously to this elephant and hoppopotamus ivory, and animal and extracted human teeth were used. The base as well as the teeth were carved from ivory. Wooden bases were sometimes used. The workmanship on these dentures were crude and the plate ill-fitting, So that Washington could justly say of his teeth that they were an infirmity. These dentures were held in place with spiral springs.

Tooth extraction was the principal part of dentistry and was performed by the family doctor or barber. The turkney was the principal instrument used and the doctor, with a red bandana handkerchief, which was not always clean, with which he made a sort of cushion for the key turned the teeth out of many a suffering mortal.

In 1766 Mr. John Woofendale was the first educated dentist in this country, having been instructed by Thomas Bedmore, dentist to Geo. III. He practiced in New York and Philadelphia and then returned to England.

The fathers and founders of American dentistry were Joseph Lemaire, a Frenchman, who was quite successful in transplanting teeth. He instructed some two or three persons. This was the first commencement in this country of educated dentists. Isaac Greenwood and son, John Greenwood, were the first dentists in Boston. John constructed the first artificial denture for George Washington. This was somewhere about 1784 or 1785. Josiah Flagg, one of Lemaire's pupils, began business as an itinerant, and was properly our first American dentist. Dr. Flagg transplants teeth, cures ulcers and eases them from pain without drawing, fastens those that are loose, mends teeth with foil or gold to be as lasting and useful as sound teeth and without pain in the operation, and secures them in a lasting, independent and serviceable manner; sews up hair lips, and fixes gold roofs and plates, greatly assisting pronunciation and swallowing; cuts the defect from teeth, restores them to whiteness and soundness, without saws, files, acids, and such abusives as have shamefully crept into the profession, and have destroyed the confidence of the public. Sells by wholesale and retail, dentrifices tinctures, chewsticks, masticks, teeth and gum brushes, suitable for every age, complaint and climate, with directions for use.

James Gardette practiced in Philadelphia. He was one of the best early American dentists, he practiced forty-five years. The first plate with atmospheric pressure as a principle is attributed to him. He substituted gold foil for lead in the filling of teeth.

Dr. John Greenwood was the first, so far as is known, to pierce the maxillary sinus. He did this by removing a molar tooth and made the cavity or opening through its socket, and thus treated the disease and cured it. He became celebrated as a surgeon, and is supposed to be the first in the United States to strike up a gold plate for the base of an artificial denture. He is best known as being the dentist to the President of the United States, George Washington. He made several sets for this eminent gentleman, one of which is now in the possession of Dr. John Allen of New York. This gentleman wore as a watch charm the last natural tooth of George Washington.

Time would fail us to speak of all the grand worthies who have contributed to make our profession what it is. We will just name them and hurry on. Horace Hayden, who published many papers on dentistry and gave a course of lectures to the medical students of the University of Maryland on Dentistry. He petitioned the legislature of Maryland for a dental college with several others. He was located at Baltimore in 1804.

Edward Hudson, who practiced in Philadelphia, attained great eminence in the profession, and proved by his excellent work that the natural teeth could be saved. E. Parmley said we are more indebted to him than any other man, for his success and the importance attached to saving the natural teeth.

To John Randall, of Boston, we are indebted for the skill which he displayed in the turnkey and forceps, long before they were articles of merchandise.

Lemard Keocher, of Philadelphia, became so eminent in practice that his income was about $8,000 per annum. He picked up the business. His first case in Philadelphia, of simple extraction, occasioned him much embarrassment. He grasped the tooth with an instrument, shut his eyes, and turning his head from the patient made a strong effort which dislodged the tooth—he being mean while under such excitement that he knew not whether the tooth was out or the jaw broken. The patient assured him he had never had a tooth extracted so easy before. Keocher was so pleased at this triumph that he dates his snccess from this point. He published a work on Dental Surgery in London, 1826, and his treatment of the dental pulp has been accepted by the best authorities. From having no teachers he became a teacher, and is worthy of great credit and honor.

The growth of inventions and dental materials has been very great in the last century and our knowledge of theory and practice has had an equally healthy growth. From the most meagre beginnings great improvements have sprung. The first teeth were human teeth, animal teeth, those of cattle and sheep, hippopotamus' tusks and teeth, elephant and other ivories, bone, etc. There were serious objections to all these, and then came the porcelain, which were plain white teeth, then the shading to match the natural teeth, with plain pins, which were bent, and the pins with heads. Many dentists displayed much art and skill in the early carved teeth. As it would well repay any one to read the history and growth of porcelain teeth.

Dr. Elias Wildman, of Philadelphia, began his experiments in 1837. He produced such results in life-like appearance as had not been obtained before and remain unexcelled to the present time. His principal success was in uniformity of gum enamel, to him must be the honor for reducing the manufacture of teeth to a scientific basis. We all know where the best teeth can be found at the present time. Bases have been of platinum, aluminum, tin, silver, gold, rubber and celluloid.

To Dr. Bean belongs the credit of the aluminum base, 1886. Dr. Geo, E. Hawes the poured tin base, 1850. To Edwin Truman the gutta percha base. To Nelson Goodyear the vulcanite base, 1851. To Messrs. I. S. Hyatt and J. W. Hyatt, celluloid, 1870. To M. L. Loomis' porcelain base, 1854. We all know the comparative value of all these and the survival of the fittest gold, platinum, vulcanite and celluloid. The methods of retaining dentures in the mouth by ligatures, clasps, springs and atmospheric adhesion will not require extended statement. The methods of pivoting we are all familiar with. And the best methods are the ones that live.

In taking impressions wax was the first used, then plaster and compounds. Swaging dies were made of zinc, lead and tin, the former being used.

The history of operative dentistry would take more time than our paper could do justice to.

Filling materials and instruments all have their inventors and special advocates, and all have some claim to notice if not merit.

The formation of dental societies and the establishment of schools to teach dentistry would all claim a share of recognition in this paper, which time will not permit.

The history of Anæsthetics is very interesting as telling how Dr. Wells, of Hartford, in 1844 got his hint, which led to the new era in tooth pulling by nitrous oxide. Sulphuric ether, as introduced by Jackson and Morton, together with chloroform, are two of the anæsthetics that made their advent about the same time and have proved a blessing to the race. Local anæsthetics have some claim to notice but time forbids a history of them now.

The materia medica of dentistry has steadily grown from a few remedies up into the hundreds, and every dentist has a few standbys and lets the world wag on.

Extraction, transplantation and replantation have their advocates, and as the problem is not solved I will leave them as an open question, simply stating that the first two are among the first steps of dentistry.

In this paper I have been only able to casually look along the line of growth and see that as a whole it has been sure and healthy. We have made some serious mistakes and have taken some backward steps and have had many failures, but in spite of all this we are making advanced strides and are becoming a more learned and scientific profession.

Forty years ago we had no dental schools now we number nearly as many schools as years named. We have the best facilities that the ingenious brains of all nations can produce.

In many states we have dental legislation and laws. We have good dental literature and periodicals, and if we can believe the signs of the times we bid fair to be one of the leading and most useful professions. There are many worthies I have not mentioned, such as Parmley, Townsend, Harris, Arthur and a host of other good men who have gone on to get the rest of their reward. I am sorry that this paper is so fragmentary and incomplete, but from the nature of the subject it could not be otherwise. *—*Odontographic Journal.*

* The credit of this paper is due to the Academy of Dental Science (Philadelphia) for its facts —W. S. A.

